# The NLRP3 inflammasome is activated by nanoparticles through ATP, ADP and adenosine

**DOI:** 10.1038/cddis.2014.576

**Published:** 2015-02-05

**Authors:** L Baron, A Gombault, M Fanny, B Villeret, F Savigny, N Guillou, C Panek, M Le Bert, V Lagente, F Rassendren, N Riteau, I Couillin

**Affiliations:** 1 INEM, CNRS, UMR7355, University of Orleans, France; 2 INSERM U991, University of Rennes, France; 3 IGF, CNRS, UMR 5203 and INSERM U661, University of Montpellier, France

## Abstract

The NLR pyrin domain containing 3 (NLRP3) inflammasome is a major component of the innate immune system, but its mechanism of activation by a wide range of molecules remains largely unknown. Widely used nano-sized inorganic metal oxides such as silica dioxide (nano-SiO_2_) and titanium dioxide (nano-TiO_2_) activate the NLRP3 inflammasome in macrophages similarly to silica or asbestos micro-sized particles. By investigating towards the molecular mechanisms of inflammasome activation in response to nanoparticles, we show here that active adenosine triphosphate (ATP) release and subsequent ATP, adenosine diphosphate (ADP) and adenosine receptor signalling are required for inflammasome activation. Nano-SiO_2_ or nano-TiO_2_ caused a significant increase in P2Y1, P2Y2, A2_A_ and/or A2_B_ receptor expression, whereas the P2X7 receptor was downregulated. Interestingly, IL-1*β* secretion in response to nanoparticles is increased by enhanced ATP and ADP hydrolysis, whereas it is decreased by adenosine degradation or selective A2_A_ or A2_B_ receptor inhibition. Downstream of these receptors, our results show that nanoparticles activate the NLRP3 inflammasome via activation of PLC-InsP3 and/or inhibition of adenylate cyclase (ADCY)-cAMP pathways. Finally, a high dose of adenosine triggers inflammasome activation and IL-1*β* secretion through adenosine cellular uptake by nucleotide transporters and by its subsequent transformation in ATP by adenosine kinase. In summary, we show for the first time that extracellular adenosine activates the NLRP3 inflammasome by two ways: by interacting with adenosine receptors at nanomolar/micromolar concentrations and through cellular uptake by equilibrative nucleoside transporters at millimolar concentrations. These findings provide new molecular insights on the mechanisms of NLRP3 inflammasome activation and new therapeutic strategies to control inflammation.

The inflammasome is a major factor of the innate immune system acting as a multiprotein platform to activate caspase-1. We showed recently that nanoparticles of TiO_2_ (nano-TiO_2_) and SiO_2_ (nano-SiO_2_) are sensed by the NLRP3 inflammasome to induce the release of mature IL-1*β*,^1^ as observed previously with the environmental irritants asbestos or silica.^2^ Despite the identification and characterisation of numerous sterile or microbial activators, the precise mechanisms mediating NLRP3 inflammasome activation remain to be determined. Here, we investigated whether ATP release and purinergic signalling through ATP, ADP and adenosine may be involved in inflammasome activation by nanoparticles. Intracellular ATP is released after cellular stress and/or activation, and purinergic signalling has been shown to modulate inflammation and immunity.^3, 4^ In the extracellular space, ATP is rapidly hydrolysed in a stepwise manner to ADP, AMP (adenosine monophosphate) and adenosine by ectoenzymes.^4^ Adenosine is then irreversibly hydrolysed to inosine by adenosine deaminase (ADA). Extracellular ATP (eATP) signals through both ATP-gated ion channels P2X and G protein-coupled receptor (GPCR) P2Y membrane receptors, whereas ADP signals through P2Y receptors and adenosine through P1 receptors (or A receptors).^5^ P2Y receptors and A receptors may be coupled to the G_q_ protein, which activates phospholipase C-beta (PLC-*β*), to the stimulatory G (G_s)_ protein, which stimulates adenylate cyclase inducing an increase in cyclic AMP (cAMP) levels, or to the G inhibitory (G_i_) protein, which inhibits adenylate cyclase. Extracellular adenosine level is the result of adenosine production from extracellular ATP and ADP, its degradation into inosine and its reuptake by cells. Both ATP and adenosine can be transported outside of the cell via diffusion or active transport, whereas only adenosine can enter the cells through adenosine transporters.^6^ Most cells possess equilibrative and concentrative adenosine transporters (respectively, ENTs and CNTs), which allow adenosine to quickly cross the plasma membrane.^7^ Intracellular adenosine is converted to ATP via phosphorylation steps mediated by adenosine kinase (AK) and AMP kinase (AMPK). The basal physiological level of extracellular adenosine has been estimated to be in the range of 30–200 nM.^8^ ATP-derived adenosine and its subsequent signalling through P1 receptors have beneficial roles in acute disease states.^4, 9^ However, during tissue injury, elevated adenosine levels participate in the progression to chronic diseases by promoting aberrant wound healing leading to fibrosis in different organs including the lungs, liver, skin and kidney. In these conditions the blockade of adenosine signalling is beneficial.^10, 11, 12, 13, 14, 15, 16^ In murine models, ADA-knockout mice present high persistent adenosine levels, which lead to airspace enlargement and fibrosis, cardinal signs of COPD and IPF.^14, 17, 18^

Here we investigate in more detail the critical contribution of purinergic signalling in driving NLRP3 inflammasome activation in response to nanoparticles pointing out the effect of ATP, ADP, as well as adenosine and its receptors. We also identify ATP-derived adenosine as a potential activator of the inflammasome.

## Results

### Nano-SiO_2_ or nano-TiO_2_ particles trigger active ATP release and IL-1*β* secretion through purinergic signalling and pannexin/connexin hemichannel activity

**Figure 1:**
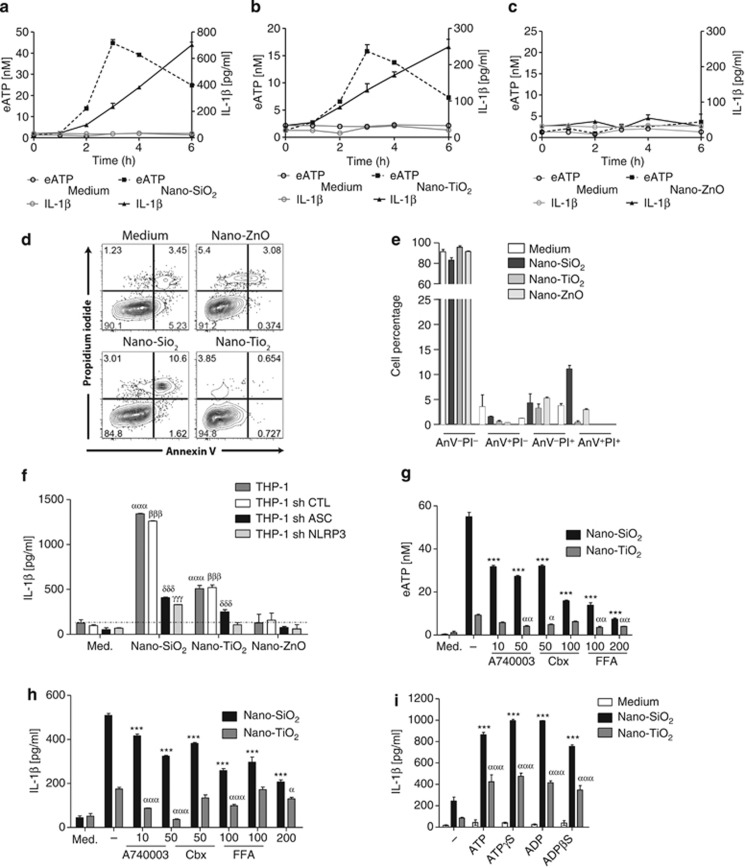
Nano-SiO_2_ or nano-TiO_2_ particles trigger active ATP release and IL-1*β* secretion through purinergic signalling and pannexin/connexin hemichannel activity. Nano-SiO_2_ (**a**) or nano-TiO_2_ (**b**) triggered active release of ATP in the supernatant by PMA-primed THP1 that peaks between 3 and 4 h. This ATP release was correlated with a secretion of IL-1*β* (**a,b**). Nano-ZnO did not induce ATP release or IL-1*β* secretion (**c**). Apoptotic (PI− anV+) and necrotic (PI+ anV−) cell death of primed THP1 was monitored using the AnnexinV/PI staining (**d**,**e**). ARL67156 (50 *μ*M) was added to the supernatant during stimulation to limit ATP catabolism (**a**–**c**). IL-1*β* secretion by nano-SiO_2_ or nano-TiO_2_ was attenuated in THP1 cells stably expressing shRNA directed against ASC (sh ASC) or NLRP3 (sh NLRP3) in comparison with THP1 transfected with lamin-specific shRNA (sh CTL) (**f**). Nano-ZnO did not induce IL-1*β* secretion after 4 h of stimulation (**f**). Specific inhibition of P2X7R by A740003 partially decreased ATP release and IL-1*β* secretion by PMA-primed THP1 after 4 h nanoparticle stimulation (**g**,**h**). Connexin/pannexin channel blocker carbenoxolone (Cbx) and connexin channel blocker flufenamic acid (FFA) reduced both ATP release and IL-1*β* secretion upon nano-SiO_2_ or nano-TiO_2_ (**g**,**h**). PMA-primed THP1 stimulated for 4 h with 200 *μ*M ATP, ADP or their stable derivatives ATP*γ*S or ADP*β*S greatly increased IL-1*β* production in response to nanoparticles, whereas these nucleotides had no effect alone (**i**). Nanoparticles are at the concentration of 250 *μ*g/ml (**a**–**h**) or 125 *μ*g/ml (**i**). Data are representative of 2–4 independent experiments. Data are mean±S.D. of triplicates, compared between untreated and nanoparticle-stimulated THP1; *ααα*, *P*≤0.001 for THP1, *βββ*, *P*≤0.001 for THP1 sh CTL, *γγγ*, *P*≤0.001 for THP1 sh ASC, *δδδ*: *P*≤0.001 for THP1 sh NLRP3 (**f).** Data are mean±S.D. of triplicates, compared between nanoparticle-stimulated THP1 and nanoparticles plus inhibitor or agonist; *** and *ααα*, *P*≤0.001 for nano-SiO_2_ and nano-TiO_2_ stimulated THP1, respectively (**g**–**i**)

**Figure 2:**
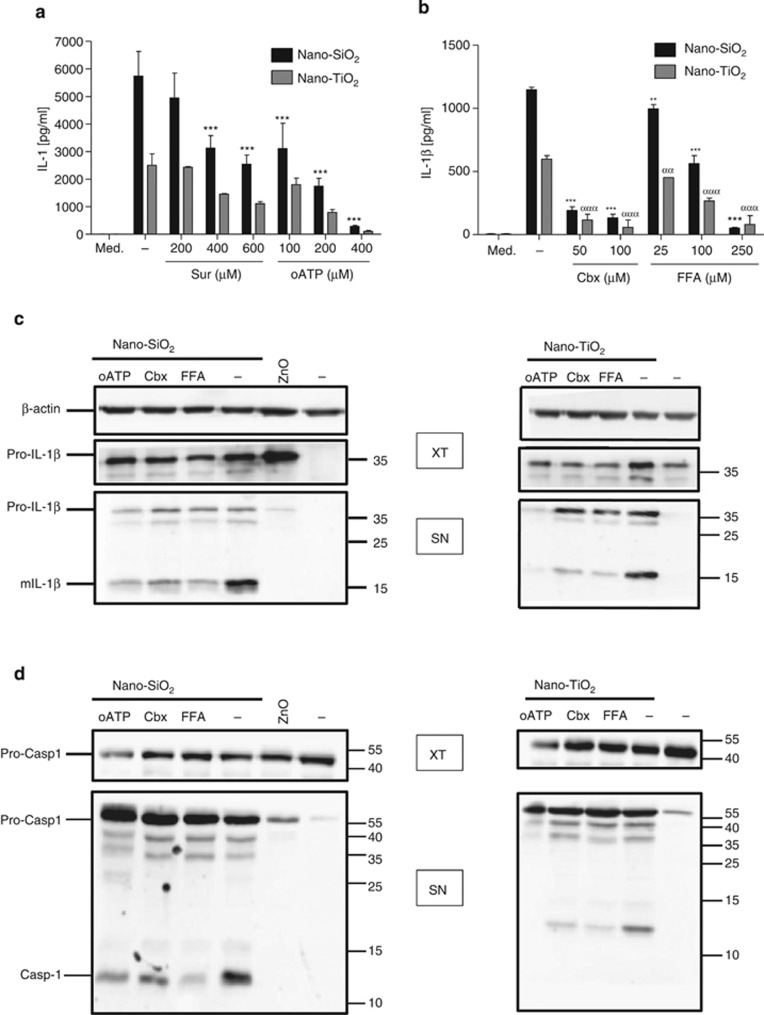
Nano-SiO_2_- or nano-TiO_2_-induced IL-1*β* in mouse macrophages is dependent on purinergic signalling. IL-1*β* production by LPS-primed BMDMs stimulated for 6 h with nano-SiO_2_ or nano-TiO_2_ was dose dependently decreased by P2R antagonists suramin (200, 400 or 600 *μ*M) and oATP (100, 200 or 400 *μ*M) (**a**). Cbx (50 or 100 *μ*M) and FFA (25, 100 or 250 *μ*M) significantly reduced IL-1*β* release by murine macrophages (**b**). Western blotting analysis of LPS-primed BMDM supernatants (SN) confirmed that FFA (100 *μ*M), Cbx (50 *μ*M) oATP (200 *μ*M) or A740003 (100 *μ*M) strongly reduced the secretion of the mature 17kD IL-1*β* form mIL-1*β* in response to nano-SiO_2_ or nano-TiO_2_ (**c**). Similarly FFA, Cbx or oATP significantly reduced autoproteolytic cleavage of the pro-caspase-1 into the secreted p10 subunit (**d**). Stimulation of BMDMs with ATP (5 mM, 45 min) induced mature IL-1*β* (**c**) and caspase-1 (**d**) releases in the supernatant, whereas nano-ZnO did not induce these cleavages (**c**,**d**). BMDM extracts (XT) prepared from the same experiments were blotted with anti-*β*-actin, anti-pro-IL-1*β* and anti-pro-caspase-1 for control (**c**,**d**). Molecular-weight markers are shown at the right (**c**,**d**). BMDMs were stimulated with 250 *μ*g/ml nanoparticles during 6 h. Data are representative of three independent experiments. Data are mean±S.D. of triplicates, compared between nanoparticle-stimulated THP1 and nanoparticles plus inhibitor; *** and *ααα,* *P*≤0.001 for nano-SiO_2_ and nano-TiO_2_ stimulated THP1, respectively (**a**,**b**)

We recently showed that nano-SiO_2_ and nano-TiO_2_, but not nano-ZnO, activate the NLRP3 inflammasome in human and murine macrophages.^1^ Here we studied whether active ATP release, purinergic signalling and connexin/pannexin channel activity are involved in inflammasome activation by nano-SiO_2_ and nano-TiO_2_. Using the ecto-ATPase inhibitor ARL67156 to limit ATP catabolism,^19^ we observed that nano-SiO_2_ (Figure 1a) or nano-TiO_2_ (Figure 1b), but not nano-ZnO (Figure 1c), causes an active release of endogenous ATP in primed THP1 macrophages, which peaks at 3–4 h and just precedes mature IL-1*β* secretion. Importantly, nano-TiO_2_, nano-SiO_2_ or nano-ZnO did not induce necrosis or apoptosis even after 6 h of stimulation (Figures 1d and e). We confirmed the importance of the inflammasome in IL-1*β* production in response to nanoparticles using THP1 cells stably expressing short hairpin ribonucleic acid (shRNA) against components of the inflammasome, the NLRP3 protein itself or the adaptor protein apoptosis-associated speck-like protein containing a CARD domain (ASC) (Figure 1f). By investigating the mechanisms of nanoparticle-induced ATP release leading to IL-1*β* secretion, we observed that specific inhibition of the P2X7 receptor (P2X7R) by A740003 at 10 *μ*M led to partial inhibition of ATP release and IL-1*β* secretion by nano-SiO_2_ and nano-TiO_2_ (Figures 1g and h). Among several potential mechanisms of nucleotide release, we focused on the connexin and pannexin families, which are able to form hemichannels.^20, 21^ The connexin/pannexin channel blockers carbenoxolone (Cbx) and flufenamic acid (FFA) significantly reduced both ATP and IL-1*β* releases (Figures 1g and h). Although unable to induce IL-1*β* by themselves, the addition of the nucleotides ATP or ADP or their stable derivatives ATP*γ*S or ADP*β*S greatly increased IL-1*β* production by THP1 cells in response to nanoparticles (Figure 1i). Unlike what we observed with THP1 human monocyte/macrophage cell line, we were unable to measure significant ATP increase in the supernatant of stimulated murine bone-marrow-derived macrophages (BMDMs). This might probably be owing to the fastest ATP degradation by these cells as proposed.^22^ However, the use of two different P2R antagonists, suramin and periodate-oxidised ATP (oATP), dose-dependently led to the reduction of IL-1*β* production induced by nano-SiO_2_ or nano-TiO_2_ (Figure 2a). Cbx and FFA also induced the reduction of IL-1*β* release (Figure 2b). Western blotting analysis confirmed that nano-SiO_2_ or nano-TiO_2_ triggers the cleavage of pro-IL-1*β* into the mature 17 kDa IL-1*β* form and its secretion in primed BMDMs. The addition of oATP, A740003, Cbx or FFA strongly reduced the secretion of mature IL-1*β* (Figure 2c). Similarly, the cleavage of pro-caspase-1 into the secreted mature p10 subunit was reduced in the presence of oATP, Cbx or FFA (Figure 2d), confirming that NLRP3 inflammasome activation depends on purinergic signalling and connexin/pannexin channels.

### Nanoparticles induce IL-1*β* secretion through metabotropic P2Y receptor signalling

**Figure 3:**
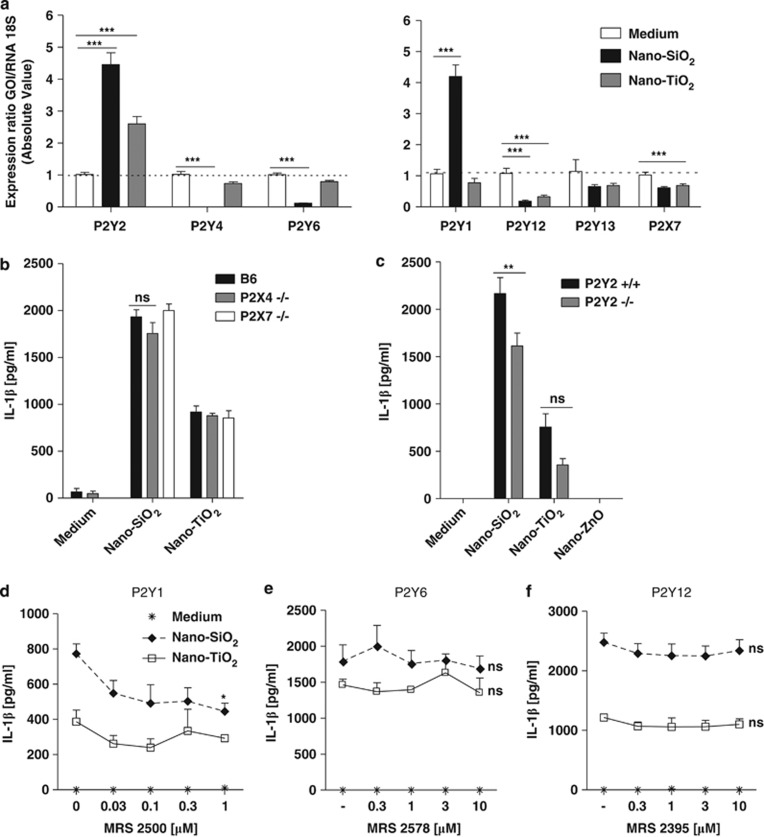
Nanoparticles induce IL-1*β* secretion through P2Y metabotropic purinergic receptors in murine macrophages. Expression of P2X7, P2Y1, P2Y2, P2Y4, P2Y6, P2Y12 and P2Y13 receptors in LPS-primed BMDMs stimulated for 4 h with nano-SiO_2_ or nano-TiO_2_ (250 *μ*g/ml) was analysed by quantitative PCR (**a**). P2Y1 (for nano-SiO_2_ only) and P2Y2 mRNA levels were increased, whereas P2X7, P2Y4, P2Y6, P2Y12 and P2Y13 mRNA levels were slightly reduced 4 h after nano-SiO_2_ or nano-TiO_2_ stimulation (**a**). Macrophages from wild-type (B6), P2X7-, P2X4- or P2Y2-deficient mice were stimulated with nanoparticles. P2X4 and P2X7 deficiency had no effect (**b**), whereas P2Y2 deficiency slightly decreased IL-1*β* secretion (**c**). IL-1*β* production by LPS-primed BMDMs was slightly decreased with the P2Y1 inhibitor MRS2500 (**d**), whereas no effect was observed with the P2Y6 inhibitor MRS2578 (**e**) and P2Y12 inhibitor MRS2395 (**f**) upon nanoparticle (250 *μ*g/ml) stimulation (Data are representative of three independent experiments *P≤0.05, **P≤0.01, ***P≤0.001, ns: not statistically different)

To identify more precisely the purinergic receptors involved, we performed quantitative mRNA expression analysis of P2 purinergic receptors. P2Y2 receptor (for ATP/UTP) mRNA level was increased after nano-SiO_2_ or nano-TiO_2_ particle stimulation, whereas P2Y1 receptor (ADP) mRNA level was increased only after nano-SiO_2_ stimulation (Figure 3a). In contrast, mRNA levels of P2Y7 (ATP), P2Y4 (UTP), P2Y6 (UDP) or P2Y12 (ADP) receptors were slightly reduced after nano-SiO_2_ stimulation (Figure 3a) and also P2Y12 receptor after nano-TiO_2_ stimulation. Deficiency in the ATP ionotropic P2X7 or P2X4 did not lead to significant impairment in IL-1*β* production by BMDMs upon nanoparticle stimulation (Figure 3b). Deficiency in the ATP/UTP metabotropic P2Y2 receptor, notably involved in cell chemotaxis in response to ATP leakage,^23^ promoted a slight decrease in IL-1*β* production (Figure 3c). In addition, we found that P2Y1 receptor antagonist MRS2500 (Figure 3d) decreased nano-SiO_2_- but not nano-TiO_2_-induced mature IL-1*β* secretion, whereas P2Y6 receptor (UDP) antagonist MRS2578 (Figure 3e) and P2Y12 receptor (ADP) antagonist MRS2395 (Figure 3f) had no effect on nanoparticle-induced IL-1*β* secretion. Altogether, these results suggest that P2Y1 (ADP) and P2Y2 (ATP/UTP) receptors are involved in the activation of the NLRP3 inflammasome by nano-SiO_2._

### Nanoparticles induce mature IL-1*β* secretion through adenosine and P1 receptors signalling

**Figure 4:**
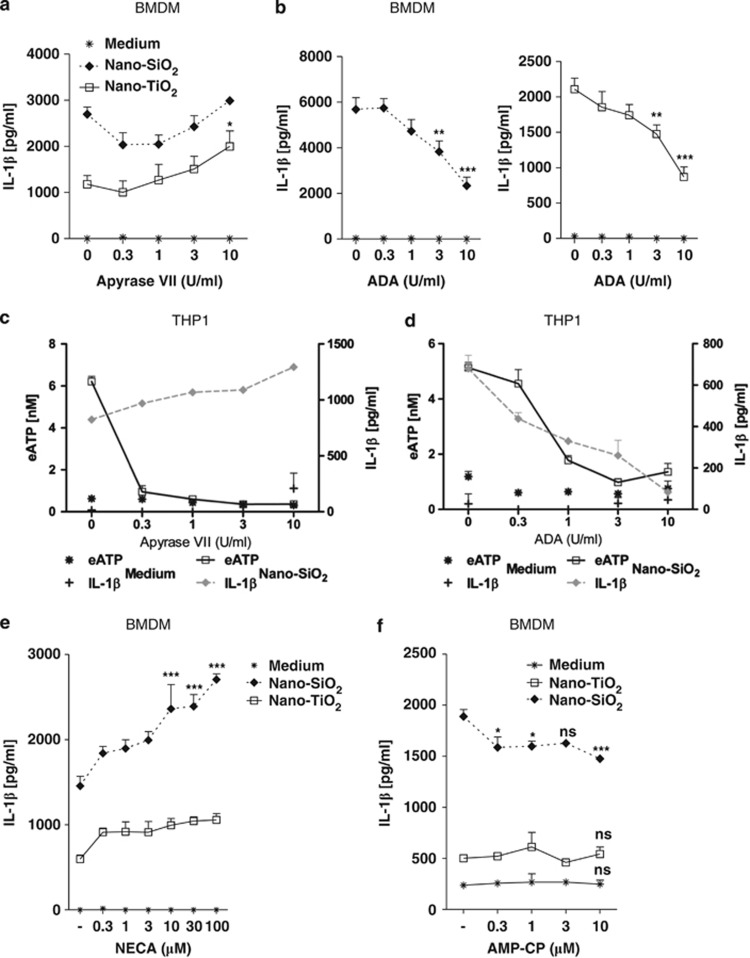
Nanoparticles induce mature IL-1*β* secretion through adenosine and P1 receptor signalling. LPS-primed murine BMDMs were stimulated for 6 h with nano-SiO_2_ or nano-TiO_2_ in the presence of increasing concentrations of apyrase grade VII (0.3, 1, 3 or 10 U/ml) (**a**). Nano-SiO_2_- and nano-TiO_2_-induced IL-1*β* were slightly decreased and then rapidly increased (**a**). LPS-primed BMDMs were also stimulated for 6 h with nano-SiO_2_ or nano-TiO_2_ in the presence of increasing doses of ADA (0.3, 1, 3 and 10 U/ml) (**b**). Apyrase grade VII or ADA alone had no effect on IL-1*β* secretion by murine macrophages (**a**,**b**). PMA-primed THP1 were stimulated for 6 h with nano-SiO_2_ (250 *μ*g/ml) in the presence of different concentrations of apyrase VII (**c**) or ADA (**d**). Nano-SiO_2_-induced eATP decreased by increasing apyrase VII, whereas IL-1*β* remained elevated (**c**). ADA dose dependently decreased both nano-SiO_2_-induced IL-1*β* and eATP secretions (**d**). Apyrase VII or ADA alone had no effect on IL-1*β* and eATP secretions by human macrophages (**c**,**d**). LPS-primed murine BMDMs were stimulated for 6 h with nano-SiO_2_ or nano-TiO_2_ in the presence of increasing concentrations of NECA (**e**) or AMP-CP (**f**). IL-1*β* secretion induced by nanoparticles was increased in the presence of NECA (**e**) and remained stable in the presence of AMP-CP (**f**). Data are representative of three independent experiments (*P≤0.05, **P≤0.01, ***P≤0.001, ns: not statistically different)

We stimulated murine macrophages in the presence of the ATP-consuming enzyme apyrase grade VII, which hydrolyses ATP and ADP into AMP. Apyrase did not abrogate IL-1*β* secretion induced by nano-TiO_2_ or nano-SiO_2_ but, on the contrary, slightly increased it (Figure 4a). Then, in the presence of the adenosine deaminase (ADA), IL-1*β* secretion by nano-SiO_2_ or nano-TiO_2_ was greatly reduced (Figure 4b). Similarly, we stimulated THP1 cells with nano-SiO_2_, in the presence of apyrase or ADA. Measurement of eATP levels showed that, even when ATP was degraded by apyrase, IL-1*β* secretion was still observed and even slightly increased (Figure 4c). When ADA was added to the nanoparticles, we noted a potent decrease in IL-1*β* and ATP levels probably owing to a shift in the balance of the ATP/ADP towards adenosine (Figure 4d). Next, the addition of the non-degradable pan-adenosine receptor agonist 5'-N-Ethylcarboxamidoadenosine (NECA; 0.3–30 *μ*M) significantly increased nano-SiO_2_- but not nano-TiO_2_-induced IL-1*β* secretion (Figure 4e). In contrast, adenosine had no effect at these concentrations, but only increase IL-1*β* at higher concentrations (100–300 mM) probably because adenosine is rapidly degraded into inosine by ADA (not shown). In addition, IL-1*β* induced by nano-SiO_2_ but not by nano-TiO_2_ was slightly decreased in the presence of the CD73 inhibitor AMP-CP, suggesting that adenosine is more important for nano-SiO_2_-induced IL-1*β* (Figure 4f). These results indicate that adenosine generated after nanoparticle-induced ATP release participates with ATP and ADP in promoting inflammation and NLRP3 inflammasome activation.

### A2_A_, A2_B_ and A3 receptors are involved in NLRP3 inflammasome activation

**Figure 5:**
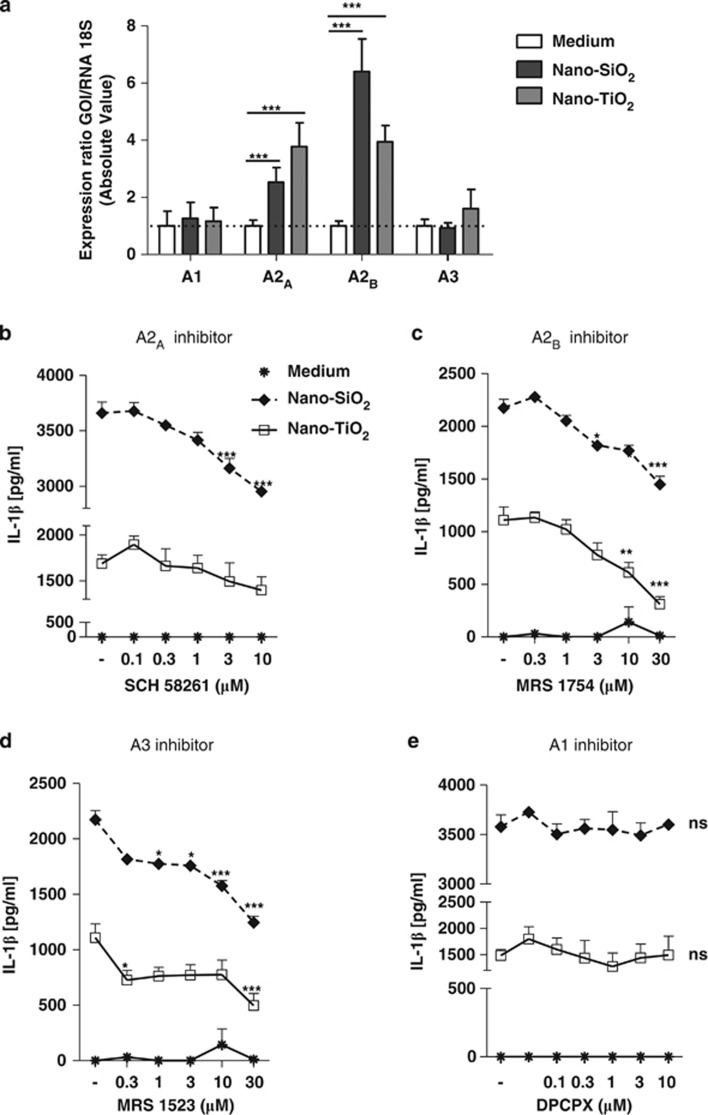
A2_A_, A2_B_ and A3 receptors were involved in NLRP3 inflammasome activation. Quantitative PCR analysis of P1 receptor expression in LPS-primed BMDMs stimulated for 4 h with nano-SiO_2_ or nano-TiO_2_. A2_A_ and A2_B_ mRNA levels were greatly increased by nanoparticles, whereas A3 mRNA was slightly increased only by nano-TiO_2_, and A1 expression remained unchanged (**a**). The specific A2_A_ (SCH58261), A2_B_ (MRS1754) and A3 (MRS1523) inhibitors dose dependently decreased IL-1*β* production by LPS-primed BMDMs, whereas specific A1 inhibitor (DPCPX) had no inhibitory effect on IL-1*β* secretion (**b-e**). Inhibitor concentrations were 0.1, 0.3, 1, 3 and 10 *μ*M for DPCPX and SCH58261, and 0.3, 1, 3, 10 and 30 *μ*M for MRS1754 and MRS1523; inhibitors alone did not induce IL-1*β* production after 6 h (**b**–**e**). Nanoparticles were used at 200–250 and 300 *μ*g/ml for nano-SiO_2_ and nano-TiO_2_, respectively. Data are representatives of 2–4 independent experiments (*P≤0.05, **P≤0.01, ***P≤0.001, ns: not statistically different)

P1 purinergic receptors mRNA expression showed that both A2_A_ and A2_B_ mRNAs are increased in the presence of nano-SiO_2_ or nano- TiO_2_, whereas A3 and A1 mRNA expression levels did not significantly change (Figure 5a). Moreover, we showed that the specific A2_A_ (SCH58261), A2_B_ (MRS1754) or the specific A3 (MRS1523) inhibitors decreased IL-1*β* secretion after nano-SiO_2_ or nano-TiO_2_ stimulation (Figures 5b–d). In contrast, the specific antagonist of A1 receptor (DPCPX) had no effect on IL-1*β* secretion (Figure 5e). These results identified adenosine as a crucial mediator of IL-1*β* secretion through the high-affinity A2_A_ receptor and the low-affinity A2_B_ and A3 receptors in response to nanoparticle activation in murine macrophages.

### Nanoparticles trigger NLRP3 inflammasome through the activation of PLC-InsP3 and inhibition of ADCY-cAMP pathways

**Figure 6:**
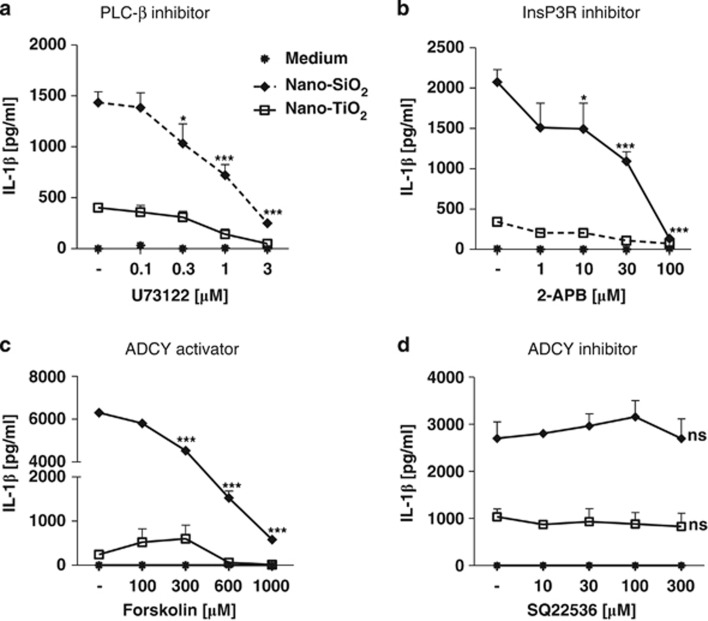
Nanoparticles trigger NLRP3 inflammasome through activation of PLC-InsP3 and inhibition of ADCY-cAMP pathways. LPS-primed murine BMDMs were stimulated for 6 h with nano-SiO_2_ or nano-TiO_2_ in the presence of the PLC-*β* inhibitor U73122 (**a**), the chelating molecule 2-APB, which blocks iCa^2+^ increase (**b**), the ADCY activator forskolin (**c**) or the ADCY inhibitor SQ22536 (**d**). IL-1*β* production was measured by ELISA. Data are representative of 2–3 independent experiments (*P≤0.05, **P≤0.01, ***P≤0.001, ns: not statistically different)

We investigated pathways leading to inflammasome activation downstream of purinergic receptors. Both P2Y and P1 receptors belong to the GPCR family acting through numerous signalling cascades and have been linked to inflammation.^24^ P2Y1, P2Y2, A3 and A2_B_ receptors involved in nanoparticle-mediated inflammasome activation can be coupled to the heterotrimeric G proteins of the G_q_ family that activate phospholipase C-*β* (PLC-*β*). We show that the inhibitor of PLC-*β*, U73122, blocked nanoparticle-induced IL-1*β* secretion (Figure 6a). PLC-*β* is able to hydrolyse phosphatidylinositol-4, 5-bisphosphate into diacylglycerol (DAG), activating the protein kinase C and the production of the inositol trisphosphate (InsP3), which in turn causes an increase in cytosolic Ca^2+^ by binding to InsP3 receptors located in the endoplasmic reticulum. As intracellular Ca^2+^ (iCa^2^) increase was shown to directly activate the NLRP3 inflammasome,^25^ we analysed the effect of 2-APB, a molecule chelating and hence blocking the increase of iCa^2+^. We observed that 2-APB strongly reduced nanoparticle-induced IL-1*β* secretion (Figure 6b). Moreover, adenosine receptors can also be coupled to the Gs family activating ADCY or the G_i/o_ family inhibiting ADCY with subsequent augmentation or reduction of cyclic AMP (cAMP). As cAMP was shown to bind and suppress NLRP3 inflammasome activation directly,^25^ we examined the involvement of ADCY in nanoparticle-induced IL-1*β* secretion. The addition of the ADCY activator forskolin dose dependently inhibited nanoparticle-induced IL-1*β* secretion (Figure 6c), whereas the addition of the ADCY inhibitor SQ22536 had no effect (Figure 6d). Collectively, these results indicate that nanoparticles trigger the NLRP3 inflammasome pleonasm through both activation of PLC-InsP3 and inhibition of ADCY-cAMP pathways.

### Adenosine induces IL-1*β* secretion and ATP release in THP1 human macrophages

**Figure 7:**
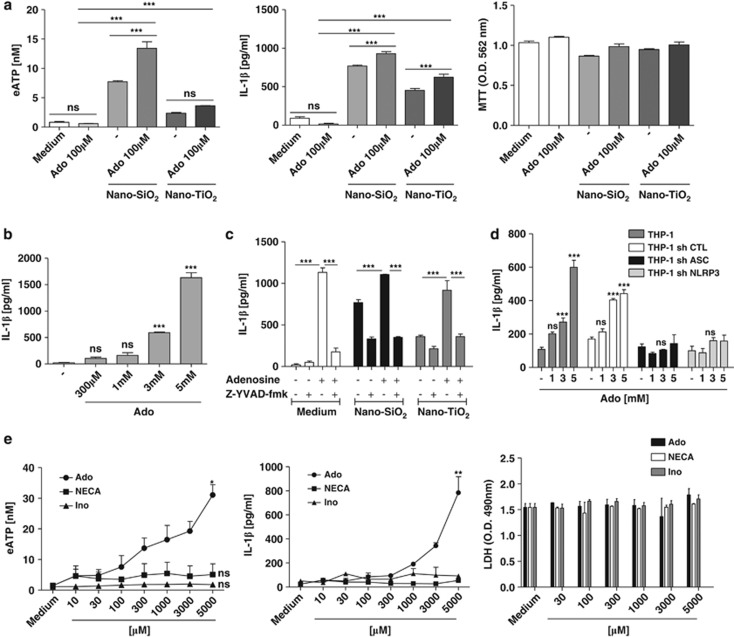

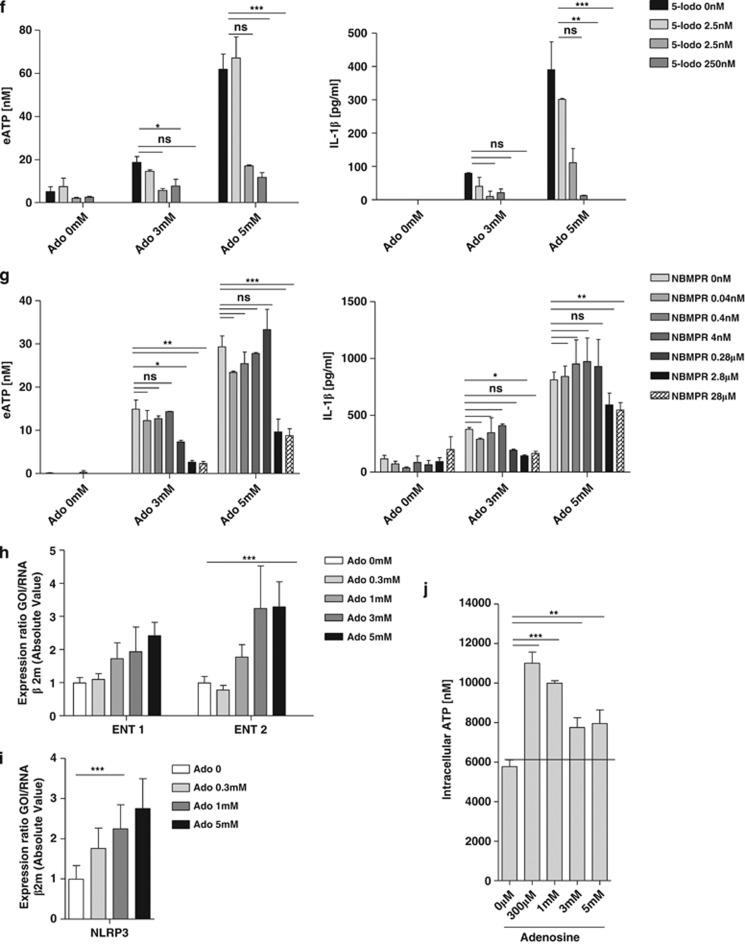
Adenosine induces IL-1*β* secretion and ATP release in THP1 human macrophages. PMA-primed THP1 cells were stimulated during 6 h with 250 *μ*g/ml nano-SiO_2_, 500 *μ*g/ml nano-TiO_2_ and/or high concentrations of adenosine (Ado), and eATP and IL-1*β* releases were measured. Adenosine potentiated eATP release and IL-1*β* secretion upon nanoparticle stimulation without inducing change in cell viability (**a**). High doses of adenosine alone induced IL-1*β* secretion by PMA-primed THP1 in a dose-dependent manner (**b**). The caspase-1-specific inhibitor Z-YVAD-fmk (5 *μ*M) remarkably reduced adenosine- and/or nanoparticle-induced IL-1*β* secretion; Z-YVAD-fmk alone had no effect (**c**). Adenosine-dependent induction of IL-1*β* secretion was reduced in THP1 sh NLRP3 or THP1 sh ASC but not in unmodified THP1 or THP1 sh CTL (**d**). PMA-primed THP1 cells were stimulated with increasing doses of adenosine, the non-metabolisable analogue of adenosine NECA or inosine (Ino), the product of adenosine hydrolysis by ADA, and cell supernatants were analyzed to measure eATP, IL-1β and cell death (**e**). PMA-primed THP1 cells were stimulated with mM doses of adenosine in the presence of 5-iodotubercidin (5-Iodo), an inhibitor of both adenosine kinase and ENTs, or in the presence of NBMPR, an inhibitor of ENT1 at nM doses and ENT2 at *μ*M concentrations, and eATP and IL-1*β* releases were measured at 6 h (**f**,**g**). Quantitative PCR analysis of ENT1, ENT2 and NLRP3 expression in PMA-primed THP1 stimulated for 6 h with increasing concentrations of Ado was performed (**h**,**i**). Intracellular ATP contents were measured 6 h after stimulation with high concentrations of adenosine (**j**). Data are representative of 2–3 independent experiments (*P≤0.05, **P≤0.01, ***P≤0.001, ns: not statistically different)

We observed that high concentrations of adenosine (100 *μ*M), which does not correspond to adenosine receptor affinities, enhanced ATP release and IL-1*β* secretion on nanoparticles in THP1 macrophages (Figure 7a). Moreover, adenosine at 5 mM was alone able to trigger ATP release and IL-1*β* secretion, whereas 100 *μ*M adenosine had no effect (Figure 7a). IL-1*β* secretion induced by nanoparticles plus adenosine (5 mM) or adenosine alone in the presence of the specific caspase-1 inhibitor Z-YVAD-fmk was greatly reduced (Figure 7c). Moreover, IL-1*β* secretion in response to a high dose of adenosine was not induced in THP1-expressing shNLRP3 or shASC (Figure 7d). Intracellular and extracellular adenosine levels are regulated by equilibrative nucleoside transporters (ENTs) present at the cell membrane.^7^ Cellular adenosine uptake may lead to intracellular metabolism of adenosine in ATP by adenosine kinase and subsequent release of ATP and IL-1*β* secretion.^6^ To test these possibilities, we stimulated THP1 macrophages with increasing doses of adenosine, NECA, the non-degradable analogue of adenosine or inosine, the metabolite of adenosine degradation by ADA. We observed that even if high doses of adenosine triggered ATP release and IL-1*β* secretion, the same doses of NECA had no effect, demonstrating that metabolism of adenosine is necessary for these responses (Figure 7e). One possibility for adenosine to be metabolised is its hydrolysis in inosine by ADA. Nevertheless, high doses of inosine were unable to promote eATP and IL-1*β* release (Figure 7e). Millimolar doses of adenosine, NECA or inosine did not induce cell death (Figure 7e). The other possibility is an adenosine reuptake through ENTs and intracellular metabolism of adenosine in ATP by adenosine kinase. To test this hypothesis, we measured eATP and IL-1*β* induced by millimolar concentrations of adenosine in the presence of 5-iodotubercidin, a pharmacological inhibitor of both adenosine kinase and ENTs, and showed that eATP and IL-1*β* release was reduced (Figure 7f). We used NBMPR, a pharmacological inhibitor of ENTs that is specific for ENT1 (Ki=0.4 nM) and ENT2 (ki=2.8 *μ*M) at nM and *μ*M concentrations, respectively. We observed that NBMPR inhibited eATP and IL-1*β* only at *μ*M doses (Figure 7g). The mRNA expression of ENT2 was significantly increased by millimolar concentrations of adenosine (Figure 7h). Millimolar concentrations of adenosine significantly increased NLRP3 mRNA expression, supporting the role of adenosine in NLRP3 inflammasome activation (Figure 7i). In addition, intracellular ATP contents were increased after addition of extracellular adenosine at high doses (Figure 7j). Altogether, these results indicate that extracellular adenosine when present at a high concentration is recaptured by macrophages through ENT2 transporters and metabolised in ATP by adenosine kinase leading to ATP release and NLRP3 inflammasome activation.

### Early nanoparticle-induced pulmonary inflammation depends on adenosine

**Figure 8:**
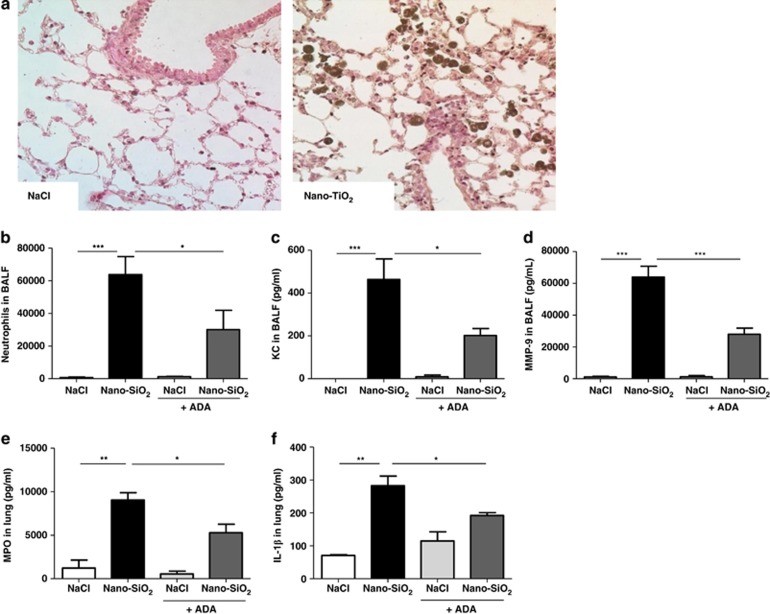
Pulmonary inflammation upon instillation of nanoparticles is partially dependent on adenosine production. C57BL/6 mice were instilled with saline solution or nano-TiO_2_ (5 mg/kg). Lung histology was performed 24 h after instillation to visualise aggregates of nanoparticles and inflammation in tissue (haematoxylin and eosin staining; original magnification, × 1000; micrographs are representatives of 5 mice per group) (**a**). Simultaneously to the instillation of vehicle or nano-SiO_2_, mice were treated or not intraperitoneally with adenosine deaminase (ADA, 5 U/mouse). Inflammation parameters were investigated at 6 h. Neutrophil counts (**b**) KC (**c**) and MMP-9 (**d**) contents were measured in the BALF. Myeloperoxidase (MPO) level (**e**) and IL-1*β* secretion (**f**) in lung homogenates were measured. Data are representative of three independent experiments. (*P≤0.05, **P≤0.01, ***P≤0.001)

Airway exposure to ultrafine particles is associated with strong infiltration of neutrophils in the airways in humans and mice.^1, 26^ We instilled mice with nano-TiO_2_ or nano-SiO_2_ and visualised the presence of nanoparticle aggregates in lung parenchyma at 24 h (Figure 8a). Similarly to nano-TiO_2_,^1^ nano-SiO_2_ elicited a considerable neutrophil influx in the BALF at 6 h (Figure 8b), which correlated with the production of the neutrophil chemoattractant KC (Figure 8c) and the metalloproteinase-9 (MMP-9) present in neutrophil *β*2 gelatinase granules (Figure 8d). Moreover, myeloperoxidase (MPO) present in neutrophils *α* azurophilic granules (Figure 8e) and IL-1*β* levels (Figure 8f) were also increased in lung homogenates. As we observed that nano-SiO_2_ instillation induced a transient increase of ATP content locally and that ATP is rapidly degraded, adenosine can act as a danger signal involved in lung inflammation. We performed local adenosine depletion experiments in mice by using ADA, which catalyses the conversion of adenosine into inosine. We observed that local treatment with ADA reduced nano-SiO_2_-induced acute inflammation, resulting in markedly reduced neutrophils (Figure 8b), KC (Figure 8c), MMP-9 (Figure 8d) contents in BALF and attenuated MPO (Figure 8e) and IL-1*β* levels (Figure 8f) in the lung. These data indicate that adenosine generated *in vivo* after nanoparticle-induced lung injury has an early pro-inflammatory role, as observed *in vitro* for macrophages.

## Discussion

Despite extensive studies, the mechanisms of NLRP3 inflammasome activation are not well understood. Here we demonstrate that SiO_2_ and TiO_2_ nanoparticles promote the secretion of mature IL-1*β* by macrophages through the active release of ATP in the extracellular space. Interestingly, ATP but also its degrading products ADP and adenosine are important signalling molecules that allow NLRP3 inflammasome activation and mature IL-1*β* secretion in macrophages.

We showed that nano-SiO_2_ and nano-TiO_2_, but not nano-ZnO, induced the active release of ATP through connexin and/or pannexin hemichannels leading to IL-1*β* secretion by macrophages. ATP release and IL-1*β* secretion depend on purinergic signalling and in particular on the P2X7R for ATP, contrarily to IL-1*β* secretion. The addition of nucleotides such as ATP or ADP, or their stable derivatives ATP*γ*S or ADP*β*S, greatly increased IL-1*β* production by macrophages, indicating that ATP and ADP are involved in nanoparticle-mediated NLRP3 inflammasome activation. Importantly, nano-SiO_2_ and/or nano-TiO_2_ increased the mRNA expression of P2Y1 and/or P2Y2, whereas P2Y4, P2Y6, P2Y12 and/or P2X7 receptor mRNAs were downregulated in primed murine macrophages. When CD39, which degrades extracellular ATP into ADP and AMP, was inhibited in THP1 cells using ARL67156, eATP and IL-1*β* levels were increased. One can hypothesise that it favours IL-1*β* secretion through the ATP-specific P2Y2 receptor. On the contrary, in the presence of adenosine deaminase (ADA), IL-1*β* was greatly but not totally reduced, pointing out an additional important role for adenosine as a major ATP-derived signalling nucleoside promoting IL-1*β* secretion after nano-SiO_2_ or nano-SiO_2_ macrophage activation. Importantly, nano-SiO_2_ and/or nano-TiO_2_ increased mRNA expression levels of A2_A_, A2_B_ and slightly of A3 receptors, but not of A1 receptors. In addition, specific A2_A_ and A2_B_ inhibitors decreased IL-1*β* secretion, confirming that adenosine is a crucial mediator of IL-1*β* secretion essentially through the high-affinity A2_A_ and the low-affinity A2_B_ adenosine receptors in response to nanoparticle activation in macrophages. In THP1 cells, adenosine degradation by ADA leads to a decrease of eATP, potentially due to the deficit of conversion of ADP/ATP from adenosine because of the increased degradation of adenosine to inosine. This reduction in ATP, ADP and adenosine allowed greatly reducing IL-1*β* secretion probably through signalling by P2Y2, P2Y1, A2_A_, A2_B_ and A3 receptors. Downstream of purinergic receptors, which are coupled to G proteins (GPCR), we propose that nanoparticles trigger maturation of IL-1*β* through activation of PLC-*β*/InsP3 and inhibition of ADCY-cAMP pathways. This suggests that intracellular Ca^2+^ increase and cAMP decrease are second signals required for NLRP3 inflammasome by the nano-SiO_2_ and nano-TiO_2._ It was shown recently that activation of another GPCR, the calcium-sensing receptor, by CaCl_2_ signals through similar pathways.^25^ In addition, we report that NECA, the non-degradable pan-adenosine receptor agonist, potentiated nanoparticle-induced IL-1*β* secretion, strengthening the role of adenosine as an essential danger signal in NLRP3 inflammasome triggering. Our work demonstrates that adenosine, as well as ATP or ADP, participates in inflammasome activation via multiple receptor signalling pathways.

**Figure 9:**
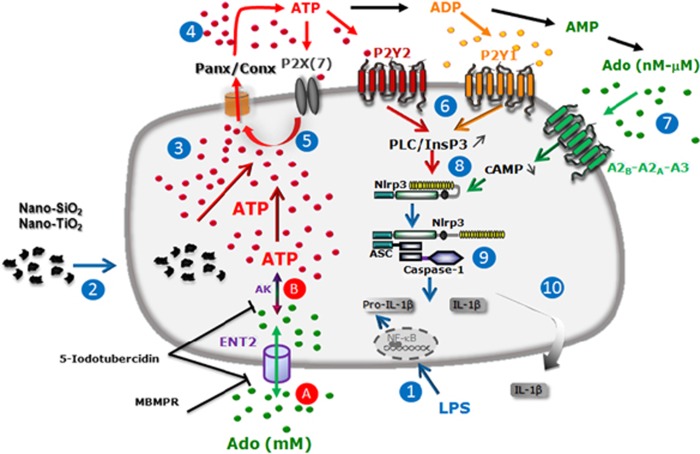
Schematic diagram illustrating the specific cascade and signalling pathway in LPS-primed macrophages stimulated with nanoparticles. LPS priming induces transcription of pro-IL-1*β* gene upon activation of the transcription factor NF-*κ*B (1). Nanoparticle uptake (2) leads to the active release of intracellular ATP (3) through pannexin/connexin hemichannels (4). This extracellular ATP (eATP) may activate ATP-gated P2X7 receptor (P2X7) to amplify ATP release in a P2X7-dependent way (5). ATP or its derived catabolism products act through on other P1 or P2 (P2X and P2Y) purinergic receptors. In particular, ATP via P2Y2 and ADP through P2Y1 activate PLC-*β*, which promotes NLRP3 inflammasome via modulation of cellular Ca^2+^ and K^+^ flux (6). Adenosine (Ado), another hydrolysed product of ATP, activates P1 receptors (A2_A_, A2_B_ and A3) leading to NLRP3 inflammasome activation (7). After NLRP3 receptor activation via signalling through multiple purinergic receptors, NLRP3 inflammasome builds up and matures pro-IL-1*β* (9) into IL-1*β*, which is secreted by macrophages (10). In case of extracellular Ado accumulation, equilibrative nucleotide transporters (ENTs) regulate adenosine through its cellular reuptake, which may be inhibited by the ENT inhibitors MBMPR and/or 5-Iodotubercidin (**a**). Metabolisation of intracellular Ado into ATP by adenosine kinase (AK) renews ATP stock (**b**) and may be inhibited by the AK inhibitor 5-Iodotubercidin. Increased intracellular ATP contents may lead to ATP release (4), NLRP3 inflammasome activation (9) and IL-1*β* secretion (10)

As extracellular ATP is degraded in adenosine within minutes, it is more likely that adenosine participates in early steps of NLRP3 inflammation activation rather than in sustained inflammasome activation, as suggested recently.^27^ Extracellular adenosine is finely regulated by adenosine degradation and cellular uptake.^28^ Nevertheless, adenosine accumulation can lead to chronic inflammation and diseases.^10^ Surprisingly, using millimolar concentrations (1–5 mM), we observed that exogenous adenosine alone was able to induce NLRP3 inflammasome activation. Adenosine receptors which affinities to adenosine range between 1 nM to 20*μ*M are probably desensitized and not involved in response to adenosine 1–5mM. Indeed, this adenosine receptor-independent effect was not mediated by millimolar doses of the non-degradable adenosine analogue, NECA, indicating that the inflammasome activation depends on adenosine metabolism and/or transport. Here, we show for the first time that exogenous adenosine at millimolar concentrations promoted NLRP3 expression and inflammasome activation and mature IL-1*β* secretion through cellular uptake and transformation into ATP by macrophages, leading to the increase of intracellular ATP content, subsequent ATP release and IL-1*β* secretion. Moreover, the functional activity of the nucleotide transporter ENT2 present at the cell membrane and of the intracellular adenosine kinase, which transforms intracellular adenosine into ATP, was required. Indeed, millimolar concentrations of adenosine were shown to efficiently increase intracellular ATP contents in primary lymphocytes and multiple cancer cell lines.^28^ In an attempt to summarise our data, we propose a model presented in Figure 9. As we showed very recently that the NLRP3 inflammasome is released as a particulate danger signal and phagocytosed by surrounding macrophages, one can imagine that adenosine uptake by these neighbouring cells may amplify the inflammatory response.^29^ Our *in vitro* results provide a new mechanism by which adenosine accumulation *in vivo* may sustain inflammasome activation, leading to chronic inflammatory diseases.^30^ Adenosine degradation in inosine by ADA could be a good strategy to attenuate adenosine-mediated inflammation via specific receptor signalling and to avoid adenosine accumulation and cellular uptake. To evaluate the role of adenosine in pulmonary inflammation and the potential anti-inflammatory effect of degradation of adenosine by ADA, we exposed mice to nano-SiO_2_ in the presence of ADA. Our results indicate that adenosine produced locally after nanoparticle exposure presents pro-inflammatory effects and that irreversible degradation of adenosine to inosine by ADA treatment reduced early pulmonary inflammation. Several studies indicated that extracellular adenosine can rise from baseline to high local concentrations in chronic diseases, adenosine being pro-inflammatory.^8, 10, 11, 12, 13, 14, 15, 16^ Our study supports the idea that adenosine can act as a pro-inflammatory mediator in certain circumstances. Moreover, we demonstrate that depending on its microenvironment concentration adenosine acts through different mechanisms, in particular adenosine-receptor signalling or adenosine cellular reuptake. Altogether, this indicates that signalling through adenosine is finely tuned and may be involved in both pro-inflammatory and anti-inflammatory processes. Nanoparticles are known to exacerbate respiratory diseases such as asthma and COPD.^31^ Their toxicity or inflammatory effects depend on nanoparticle shape and size and the amount of metal ion released.^32^ Cobalt-chromium nanoparticles were shown to induce human fibroblast damages without crossing the plasma membrane, through transmission of ATP via hemichannels and pannexin channels and intercellular signalling.^33^ Nevertheless, the role of adenosine was never described in nanoparticle-mediated damage or inflammation.

In conclusion, after tissue injury, adenosine may activate the inflammasome through membrane receptor signalling. In case of chronic inflammation, adenosine may accumulate and act via its cellular uptake and conversion into intracellular ATP, allowing amplifying and/or prolonging inflammasome activation. This may explain why sustained high adenosine levels are pro-inflammatory. Our findings may provide new therapeutic approaches to control chronic inflammation by inhibiting nucleotide receptors, nucleoside transporters and/or adenosine kinase activation.

## Methods

### Reagents

Nano-SiO_2_ and nano-TiO_2_ were purchased from IoLiTec (Heilbronn, Germany), and nano-ZnO is a gift from Dr. Amir Yazdi (Lausanne, Switzerland). Nanoparticles were sonicated for 30 min and used at a concentration of 125–500 *μ*g/ml *in vitro* or at a concentration of 5 mg/kg *in vivo,* as mentioned. A740003 is a gift from Dr. F. Rassendren (Montpellier, France). Adenosine deaminase (ADA) (A5168), ADP, ADP*β*S, adenosine (Ado), apyrase grade VII (A6535), ARL67156, ATP*γ*S, ATP, carbenoxolone (Cbx), DPCPX, flufanemic acid (FFA), inosine (Ino), MRS1523, MRS1754, MRS2395, MRS2578, periodate-oxidised ATP (oATP), phorbol 12-myristate 13-acetate (PMA), SCH58261 and U73122 were from Sigma (St. Quentin Fallavier, France); suramin was from VWR (Fontenay-sous-bois, France); LPS (lipopolysaccharide from *Escherichia coli*, serotype 055:B5) was from Invivogen (Toulouse, France); 2-APB, 5-Iodotubercidin, Forskolin, MRS2500, NBMPR and Z-YVAD-fmk were from Tocris (Bristol, UK) and NECA and SQ22536 were from Merck Millipore (Nottingham, UK).

### Mice

C57BL/6 wild-type mice were bred in our animal facility (CNRS, Orleans). The animals used were eight to ten weeks old, and they were kept in isolated and ventilated cages. All animal experiments complied with the French Government's ethical and animal experiment regulations.

### Lung inflammation model

Nano-SiO_2_ or nano-TiO_2_ in saline (5 mg/kg) or vehicle alone was administered by intranasal instillation under light ketamine (Imalgène 1000, 1.25 mg/ml) and xylazine (Rompun 0.1%) anaesthesia. Bronchoalveolar lavage (BAL) and lung tissue were assayed after 6 h. The lungs were homogenised in a solution containing 10 mM potassium phosphate and 0.1 mM EDTA (Sigma), centrifuged at 10 000 r.p.m. for 10 min and the supernatants were stored at −20 °C for further analysis. BAL was performed as previously described.^34^ Differential cell counts were performed by counting an average of 250 cells on cytospin preparations (Shandon CytoSpin 3, Thermo Scientific, Illkirch, France) after May-Grünwald-Giemsa staining (Diff Quick, Medion Diagnostics, Düdingen, Switzerland) according to the manufacturer's instructions. After BAL and lung perfusion, the large lobe was fixed and 3-*μ*m sections were stained as described previously.^35^

### ELISA

IL-1*β*, KC, MMP-9 and MPO levels were determined using ELISA assay kits (Mouse or human DuoSet, R&D system, Minneapolis, MN, USA) according to the manufacturer's instructions.

### THP1 culture and stimulation

Monocyte/macrophage THP1 cells are a gift from Dr. Amir Yazdi (Lausanne, Switzerland) and cultured in RMPI Medium 1640 (Gibco, Illkirch, France) with 10% fetal calf serum (Hyclone, Cramlington, UK) and penicillin/streptomycin (100 U/ml, Invitrogen). For experiments, THP1 were differentiated for 3 h with 0.5 *μ*M PMA, washed and plated overnight (2 × 10^5^ cells/well). Cells were stimulated for indicated times, the supernatant was collected for immediate ATP measurement and/or stored for further IL-1*β* quantification.

Cell death was monitored by MTT using a standard protocol. Thiazollyl blue tetrazolium bromide (Sigma) solution was added onto the cells after supernatant collection and incubated for 2 h at 37 °C, and a 10% SDS acetic acid solution is then added. MTT reduction to formazan was quantified by an absorbance microplate reader (EL800, BioTek, Colmar, France) at 610nm (KC4 software). Apoptotic and necrotic cell death of primed THP1 cells was also monitored using AnnexinV/PI staining (eBioscience, San Diego, CA, USA). Cells were gently detached using repeated aspiration and expulsion of cold PBS. After centrifugation, cells were resuspended in annexin V binding buffer and stained for 20 min with Annexin V and propidium iodide. Data were collected on a BD FACSCanto and analysed using Flowjo software (Tree Star, OR, USA).

### Sh RNA THP1

THP1 stably expressing short hairpin RNA (shRNA) against lamin (‘sh CTL'), ASC or NLRP3 are kind gifts from Dr. Fabio Martinon (Lausanne, Switzerland) and were obtained as previously described (Pétrilli *et al.*, 2007). shRNA THP1 cells were cultured in RPMI Medium 1640 (Gibco) with 10% fetal calf serum (Hyclone) and 4 *μ*g/ml puromycin (Gibco). Cell priming and stimulation are the same as previously described for untransfected THP1 cells.

### ATP measurement

Extracellular ATP in cell-free medium supernatant was quantified using ATP Lite one step kit (Perkin Elmer, Courtaboeuf, France) according to the manufacturer's instructions, and the luminescence produced was measured (Mithras, Mikrowin 2000 software, Berthold Technologies, Thoiry, France).

### BMDM culture and stimulation

Primary BMDMs were obtained from femoral bone marrow as described.^36^ In brief, cells from femurs of C57BL/6 mice were isolated and cultured at 10^6^ cells/ml for 7 days in Dulbecco's minimal essential medium (DMEM, Sigma) supplemented with 20% horse serum and 30% L929 cell-conditioned medium as a source of M-CSF. Three days after washing and culturing in fresh medium, the cell preparation contained a homogeneous population of >95% macrophages (Müller *et al.*^36^). The BMDMs were plated in 96-well microculture plates (2.10^5^cells/well) and stimulated with 100 ng/ml LPS during 3 h to induce pro-IL-1*β* production. Particles were applied for 6 h and cell supernatants were collected after 6 h and stored for further analysis. The absence of cytotoxicity of the different stimuli used was verified by MTT assay using the standard protocol.

### Quantitative RT-PCR

BMDM or THP1 cells were plated in six-well microculture plates (at 5 × 10^6^ cells/well), stimulated during 3 h with LPS or PMA, respectively, and were washed and stimulated with particles for 4 or 6 h. RNA was extracted using the RNeasy Mini Kit (Qiagen, Courtaboeuf, France) and particles were removed by centrifugation (10 000 g, 10 min, 4 °C). All primers were synthesised (Qiagen). The expression levels of A1, A2_A_, A2_B_, A3, P2X7, P2Y1, P2Y2, P2Y4, P2Y6, P2Y12 and P2Y13 receptor mRNAs, relative to housekeeping 18S mRNA, and expression of ENT1 and ENT2 transporter mRNAs, relative to housekeeping *β*2m mRNA, were analysed using Quantitect gene expression assays (Qiagen). Reverse transcription was performed by SuperScript III Reverse Transcriptase (Fisher Invitrogen) according to the manufacturer's instructions for amplification. RT-PCR was performed starting from 500 ng of total RNA using a Stratagene Mx3005P real-time PCR system (Agilent Technologies, Massy, France). For all experiments, biological quadruplicates and technical triplicates were performed.

### Immunoblotting

BMDM cells were plated in 12-well microculture plates (at 3 × 10^6^ cells/well), stimulated with LPS during 3 h and were washed and stimulated with particles during 6 h. Supernatants were collected and stored at −20 °C for further analysis. BMDMs were washed with cold PBS and scraped in lysis buffer solution (150 mM NaCl, 10 mM Tris pH 8, 1 mM EDTA, 0.2% SDS and 1% Nonidet P-40) supplemented with a protease inhibitor cocktail (1%) and pefabloc (0.1 mg/ml) (Roche Applied Science, Meylan, France). Lysis extracts and supernatants were collected and protein content was measured (DC protein assay, Bio-Rad, Munich, Germany). Proteins were denatured by boiling (95 °C, 5 min), separated by SDS–PAGE and transferred to nitrocellulose membranes. The membranes were immunoblotted with a primary goat anti-IL-1*β* antibody (Sigma Aldrich) or rabbit anti-caspase-1 p10 (Santa Cruz Biotechnology) and proteins were detected with appropriate secondary antibody followed by enhanced chemiluminescence (ECL, Fisher, Illkirch, France).

### Statistical analysis

Statistical evaluation of differences between the experimental groups was determined by ANOVA followed by Bonferroni's test using the Prism 5.0 software (GraphPad). *P*-values <0.05 were considered statistically significant.

## Abbreviations

ADA: adenosine deaminase
ADCY: adenylate cyclase
ADP: adenosine diphosphate
AK: adenosine kinase
AMP: adenosine monophosphate
AMPK: AMP kinase
AR: adenosine receptor/P1 receptor
ASC: apoptosis-associated speck-like protein containing a CARD domain
ATP: adenosine triphosphate
BAL: bronchoalveolar lavage
BALF: BAL fluid
BMDM: bone-marrow-derived macrophages
cAMP: cyclic AMP
Cbx: carbenoxolone
CNT: concentrative nucleotide transporter
COPD: chronic obstructive pulmonary disease
eATP: extracellular ATP
ENPPs: ecto-nucleotidase pyrophosphatases/phosphodiesterases
ENT: equilibrative nucleotide transporter
FFA: flufenamic acid
G_i_: inhibitory G protein
GPCR: G protein-coupled receptor
G_s_: stimulatory G protein
IL-1*β*: interleukin-1 beta
InsP3: inositol triphosphate
IPF: idiopathic pulmonary fibrosis
LPS: lipopolysaccharide
MMP-9: metalloproteinase-9
MPO: myeloperoxidase
MSU: monosodium urate
NLR: nod-like receptor
Nano-SiO_2_ and -TiO_2_: silica dioxide and titanium dioxide nanoparticles
NLRP3: NLR pyrin domain containing 3
oATP: periodate-oxidised ATP
PLC-*β*: phospholipase C-beta
PMA: phorbol 12-myristate 13-acetate
ROS: reactive oxygen species
sh RNA: short-hairpin ribonucleic acid.

## References

[bib1] 1Yazdi AS, Drexler SK, Tschopp J. The role of the inflammasome in nonmyeloid cells. J Clin Immunol 2010; 30: 623–627. 20582456 10.1007/s10875-010-9437-y

[bib2] 2Dostert C, Meylan E, Tschopp J. Intracellular pattern-recognition receptors. Adv Drug Deliv Rev 2008; 60: 830–840. 18280002 10.1016/j.addr.2007.12.003

[bib3] 3Antonioli L, Pacher P, Vizi ES, Hasko G. CD39 and CD73 in immunity and inflammation. Trends Mol Med 2013; 19: 355–367. 23601906 10.1016/j.molmed.2013.03.005 PMC3674206

[bib4] 4Eltzschig HK, Sitkovsky MV, Robson SC. Purinergic signaling during inflammation. N Engl J Med 2012; 367: 2322–2333. 23234515 10.1056/NEJMra1205750 PMC3675791

[bib5] 5Jacobson KA, Balasubramanian R, Deflorian F, Gao ZG. G protein-coupled adenosine (P1) and P2Y receptors: ligand design and receptor interactions. Purinergic Signal 2012; 8: 419–436. 22371149 10.1007/s11302-012-9294-7 PMC3360101

[bib6] 6Baldwin SA, Beal PR, Yao SY, King AE, Cass CE, Young JD. The equilibrative nucleoside transporter family, SLC29. Pflugers Arch 2004; 447: 735–743. 12838422 10.1007/s00424-003-1103-2

[bib7] 7Young JD, Yao SY, Baldwin JM, Cass CE, Baldwin SA. The human concentrative and equilibrative nucleoside transporter families, SLC28 and SLC29. Mol Aspects Med 2013; 34: 529–547. 23506887 10.1016/j.mam.2012.05.007

[bib8] 8Chen JF, Eltzschig HK, Fredholm BB. Adenosine receptors as drug targets—what are the challenges? Nat Rev Drug Discov 2013; 12: 265–286. 23535933 10.1038/nrd3955 PMC3930074

[bib9] 9Eckle T, Fullbier L, Wehrmann M, Khoury J, Mittelbronn M, Ibla J et al. Identification of ectonucleotidases CD39 and CD73 in innate protection during acute lung injury. J Immunol 2007; 178: 8127–8137. 17548651 10.4049/jimmunol.178.12.8127

[bib10] 10Karmouty-Quintana H, Xia Y, Blackburn MR. Adenosine signaling during acute and chronic disease states. J Mol Med (Berl) 2013; 91: 173–181. 23340998 10.1007/s00109-013-0997-1 PMC3606047

[bib11] 11Huszar E, Vass G, Vizi E, Csoma Z, Barat E, Molnar Vilagos G et al. Adenosine in exhaled breath condensate in healthy volunteers and in patients with asthma. Eur Respir J 2002; 20: 1393–1398. 12503694 10.1183/09031936.02.00005002

[bib12] 12Esther CR Jr., Lazaar AL, Bordonali E, Qaqish B, Boucher RC. Elevated airway purines in COPD. Chest 2011; 140: 954–960. 21454402 10.1378/chest.10-2471 PMC3186686

[bib13] 13Goodarzi MT, Abdi M, Tavilani H, Nadi E, Rashidi M. Adenosine deaminase activity in COPD patients and healthy subjects. Iran J Allergy Asthma Immunol 2010; 9: 7–12. 20548128

[bib14] 14Chunn JL, Molina JG, Mi T, Xia Y, Kellems RE, Blackburn MR. Adenosine-dependent pulmonary fibrosis in adenosine deaminase-deficient mice. J Immunol 2005; 175: 1937–1946. 16034138 10.4049/jimmunol.175.3.1937

[bib15] 15Sun CX, Zhong H, Mohsenin A, Morschl E, Chunn JL, Molina JG et al. Role of A2B adenosine receptor signaling in adenosine-dependent pulmonary inflammation and injury. J Clin Invest 2006; 116: 2173–2182. 16841096 10.1172/JCI27303 PMC1501110

[bib16] 16Zaynagetdinov R, Ryzhov S, Goldstein AE, Yin H, Novitskiy SV, Goleniewska K et al. Attenuation of chronic pulmonary inflammation in A2B adenosine receptor knockout mice. Am J Respir Cell Mol Biol 2010; 42: 564–571. 19556606 10.1165/rcmb.2008-0391OC PMC2874442

[bib17] 17Aldrich MB, Blackburn MR, Datta SK, Kellems RE. Adenosine deaminase-deficient mice: models for the study of lymphocyte development and adenosine signaling. Adv Exp Med Biol 2000; 486: 57–63. 11783528 10.1007/0-306-46843-3_11

[bib18] 18Sun CX, Young HW, Molina JG, Volmer JB, Schnermann J, Blackburn MR. A protective role for the A1 adenosine receptor in adenosine-dependent pulmonary injury. J Clin Invest 2005; 115: 35–43. 15630442 10.1172/JCI22656 PMC539198

[bib19] 19Joseph SM, Buchakjian MR, Dubyak GR. Colocalization of ATP release sites and ecto-ATPase activity at the extracellular surface of human astrocytes. J Biol Chem 2003; 278: 23331–23342. 12684505 10.1074/jbc.M302680200

[bib20] 20Harris AL. Connexin channel permeability to cytoplasmic molecules. Prog Biophys Mol Biol 2007; 94: 120–143. 17470375 10.1016/j.pbiomolbio.2007.03.011 PMC1995164

[bib21] 21Orellana JA, Froger N, Ezan P, Jiang JX, Bennett MV, Naus CC et al. ATP and glutamate released via astroglial connexin 43 hemichannels mediate neuronal death through activation of pannexin 1 hemichannels. J Neurochem 2011; 118: 826–840. 21294731 10.1111/j.1471-4159.2011.07210.x PMC3108012

[bib22] 22Cohen HB, Briggs KT, Marino JP, Ravid K, Robson SC, Mosser DM. TLR stimulation initiates a CD39-based autoregulatory mechanism that limits macrophage inflammatory responses. Blood 2013; 122: 1935–1945. 23908469 10.1182/blood-2013-04-496216 PMC3772500

[bib23] 23Chen Y, Corriden R, Inoue Y, Yip L, Hashiguchi N, Zinkernagel A et al. ATP release guides neutrophil chemotaxis via P2Y2 and A3 receptors. Science 2006; 314: 1792–1795. 17170310 10.1126/science.1132559

[bib24] 24Sun L, Ye RD. Role of G protein-coupled receptors in inflammation. Acta Pharmacol Sin 2012; 33: 342–350. 22367283 10.1038/aps.2011.200 PMC4085652

[bib25] 25Lee GS, Subramanian N, Kim AI, Aksentijevich I, Goldbach-Mansky R, Sacks DB et al. The calcium-sensing receptor regulates the NLRP3 inflammasome through Ca2+ and cAMP. Nature 2012; 492: 123–127. 23143333 10.1038/nature11588 PMC4175565

[bib26] 26Nordenhall C, Pourazar J, Blomberg A, Levin JO, Sandstrom T, Adelroth E. Airway inflammation following exposure to diesel exhaust: a study of time kinetics using induced sputum. Eur Respir J 2000; 15: 1046–1051. 10885423 10.1034/j.1399-3003.2000.01512.x

[bib27] 27Ouyang X, Ghani A, Malik A, Wilder T, Colegio OR, Flavell RA et al. Adenosine is required for sustained inflammasome activation via the A(2)A receptor and the HIF-1alpha pathway. Nat Commun 2013; 4: 2909. 24352507 10.1038/ncomms3909 PMC3895487

[bib28] 28Li S, Li X, Guo H, Liu S, Huang H, Liu N et al. Intracellular ATP concentration contributes to the cytotoxic and cytoprotective effects of adenosine. PLoS One 2013; 8: e76731. 24098558 10.1371/journal.pone.0076731 PMC3789704

[bib29] 29Baroja-Mazo A, Martin-Sanchez F, Gomez AI, Martinez CM, Amores-Iniesta J, Compan V et al. The NLRP3 inflammasome is released as a particulate danger signal that amplifies the inflammatory response. Nat Immunol 2014; 15: 738–748. 24952504 10.1038/ni.2919

[bib30] 30Lu Q, Newton J, Hsiao V, Shamirian P, Blackburn MR, Pedroza M. Sustained adenosine exposure causes lung endothelial barrier dysfunction via nucleoside transporter-mediated signaling. Am J Respir Cell Mol Biol 2012; 47: 604–613. 22744860 10.1165/rcmb.2012-0012OC PMC3547102

[bib31] 31Hussain S, Vanoirbeek JA, Luyts K, De Vooght V, Verbeken E, Thomassen LC et al. Lung exposure to nanoparticles modulates an asthmatic response in a mouse model. Eur Respir J 2011; 37: 299–309. 20530043 10.1183/09031936.00168509

[bib32] 32Raghunathan VK, Devey M, Hawkins S, Hails L, Davis SA, Mann S et al. Influence of particle size and reactive oxygen species on cobalt chrome nanoparticle-mediated genotoxicity. Biomaterials 2013; 34: 3559–3570. 23433773 10.1016/j.biomaterials.2013.01.085

[bib33] 33Bhabra G, Sood A, Fisher B, Cartwright L, Saunders M, Evans WH et al. Nanoparticles can cause DNA damage across a cellular barrier. Nat Nanotechnol 2009; 4: 876–883. 19893513 10.1038/nnano.2009.313

[bib34] 34Couillin I, Vasseur V, Charron S, Gasse P, Tavernier M, Guillet J et al. IL-1R1/MyD88 signaling is critical for elastase-induced lung inflammation and emphysema. J Immunol 2009; 183: 8195–8202. 20007584 10.4049/jimmunol.0803154

[bib35] 35Gasse P, Mary C, Guenon I, Noulin N, Charron S, Schnyder-Candrian S et al. IL-1R1/MyD88 signaling and the inflammasome are essential in pulmonary inflammation and fibrosis in mice. J Clin Invest 2007; 117: 3786–3799. 17992263 10.1172/JCI32285 PMC2066195

[bib36] 36Muller M, Eugster HP, Le Hir M, Shakhov A, Di Padova F, Maurer C et al. Correction or transfer of immunodeficiency due to TNF-LT alpha deletion by bone marrow transplantation. Mol Med 1996; 2: 247–255. 8726467 PMC2230110

